# A randomized, blinded, controlled and multi-centered field study comparing the efficacy and safety of Bravecto™ (fluralaner) against Frontline™ (fipronil) in flea- and tick-infested dogs

**DOI:** 10.1186/1756-3305-7-83

**Published:** 2014-03-04

**Authors:** Nadja Rohdich, Rainer KA Roepke, Eva Zschiesche

**Affiliations:** 1 MSD Animal Health Innovation GmbH, Zur Propstei, 55270 Schwabenheim, Germany

**Keywords:** Fleas, Ticks, Bravecto™ (fluralaner), Isoxazoline, Frontline™ (fipronil), Efficacy, Field study, Dog

## Abstract

**Background:**

Fluralaner, a new molecular entity of the isoxazoline class, has potent insecticidal and acaricidal activity and can be safely administered orally to dogs.

**Methods:**

A randomized, investigator-blinded, multi-centered field study compared the flea- and tick-control efficacy for dogs over a 12-week period with either a single oral dose of Bravecto™ (fluralaner) formulated as a chewable tablet or with three sequential topical Frontline™ (fipronil) treatments. Individual dogs were the experimental unit for ticks and households were the experimental unit for fleas. A total of 108 tick-infested dogs were treated with Bravecto™ (fluralaner) and 54 tick-infested dogs were treated with Frontline™ (fipronil). Dogs in 115 flea-infested households received Bravecto™ (fluralaner) and dogs in 61 flea-infested households received Frontline™ (fipronil). Flea and tick counts were conducted on all dogs at weeks 2, 4, 8, and 12 following initial treatment and efficacy was calculated as the mean percent reduction in tick or flea count at each time point compared with the mean pretreatment initiation count for each treatment group. Additionally, the percentages of tick-free and flea-free households were determined.

**Results:**

At weeks 2, 4, 8, and 12, Bravecto™ (fluralaner) flea-control efficacy in treated households was 99.2%, 99.8%, 99.8%, and 99.9% respectively, while Frontline™ (fipronil) efficacy was 94.1%, 93.0%, 96.0%, and 97.3%, respectively. Bravecto™ (fluralaner) tick-control efficacy on treated dogs at weeks 2, 4, 8, and 12 was 99.9%, 99.9%, 99.7%, and 100%, respectively, and Frontline™ (fipronil) tick efficacy was 97.6%, 93.8%, 100%, and 100%, respectively. Of dogs showing clinical flea allergy dermatitis (FAD) signs at the study start, 85.7% in the Bravecto™ (fluralaner)-treated group and 55.6% in the Frontline™ (fipronil)-treated group were evaluated at each time point as showing no clinical signs of FAD until study completion.

**Conclusions:**

Bravecto™ (fluralaner) administered once orally to dogs in a chewable tablet was highly effective for 12 weeks against fleas and ticks on privately-owned dogs and was significantly non-inferior (ticks) and superior (fleas) in comparison with topical Frontline™ (fipronil) administered 3 times sequentially.

## Background

New and effective treatments of companion animal ectoparasite infestations are important because these parasites can become tolerant to existing treatment options and have the potential to develop resistance [1]. Veterinarians and animal owners are also looking for more effective and longer lasting treatments to include in their ectoparasite control protocols. They have concerns about the irritation and injury caused directly by fleas and ticks and the risks these parasites introduce as infectious-disease vectors. Additionally, poor owner compliance is a concern with current monthly ectoparasite control retreatment recommendations [2].

Fluralaner is a member of the isoxazoline class, a novel class of antiparasitic drugs representing safe and effective new acaricidal and insecticidal products for management of ectoparasitic infestations on dogs and cats [3]. Fluralaner has been proven to have potent efficacy against ectoparasites and to be safe for oral administration to dogs [4]. *In vitro* testing determined that fluralaner is a highly potent arthropod-specific GABA-gated chloride channel inhibitor, with a less potent, but still significant, inhibitory activity on arthropod glutamate-gated chloride channels and its receptor binding was 5–236 fold better than fipronil on arthropod GABA-gated chloride channels [5]. This receptor potency difference could translate into improved efficacy for fluralaner over fipronil for ectoparasite control under field conditions.

This study presents results of a rigorous, Good Clinical Practice (GCP) compliant, blinded field study comparing Bravecto™ (fluralaner) with a positive control (fipronil) under typical veterinary practice conditions. This study evaluated whether Bravecto™ (fluralaner) is statistically at least as effective (non-inferiority analysis) as the positive control treatment.

## Methods

This was a multicenter, randomized, field efficacy study conducted at veterinary practices in Germany, France, and Spain, which enrolled privately-owned dogs of any breed or gender. The study started in August 2011 and was finished in February 2012. The veterinarians who performed clinical assessments and parasite counts were blinded to the treatment allocation of the dogs. The study design used the individual dog as the experimental unit for statistical analysis of tick infestations and the household as the experimental unit for analysis of flea infestations.

For enrollment in the study, dogs had to have either a visible flea or tick infestation (or both) on initial examination, with observation of at least 4 individual ectoparasites. The dogs also needed to have an appropriate temperament to permit required manipulations for parasite counting; to be 10 weeks or older, at least 2 kg body weight, and sufficiently healthy to follow the study schedule. Dogs were ineligible if the household included a severely ill animal requiring intensive veterinary care, any dog who had received previous ectoparasiticide treatment within the previous 7 to 30 days depending on the expected duration of effect of the treatment, a pregnant or lactating female dog, more than 5 dogs, or other pet species that could harbor fleas and/or ticks (e.g. cats, rabbits, guinea pigs) sharing the same resting area; if insecticide or insect growth regulator had been applied in the household environment within the previous two months; or if the dog would need to spend substantial time at a dog sitter or animal shelter during the study period. Dog owners were briefed on the study protocol and required to sign an informed consent allowing enrollment of their dog(s) in the study.

Dogs were randomly assigned, using a computer-generated list, to receive either Bravecto™ (fluralaner, 25–56 mg/kg body weight once) or a positive control treatment Frontline™ (fipronil, ≥6.7 mg/kg for three sequential times) at a 2:1 fluralaner:fipronil enrollment ratio. All dogs from the same household were treated using the same product. A clinical examination including a descriptive evaluation of skin and hair examinations to document any skin lesions possibly related to FAD was completed. An initial parasite count was performed using the World Association for the Advancement of Veterinary Parasitology (WAAVP) comb-counting method [6] followed by administration of the initial treatment in the veterinary hospital. All dogs that received the oral Bravecto™ (fluralaner) treatment were initially offered the opportunity to eat the tablet voluntarily. If the tablet was refused, the dogs were given the tablet directly into the mouth.

All dogs remained in the owner’s home and were fed their usual diet with access to water according to their normal routine. Grooming, bathing, and swimming were permitted during the study, but not within 3 days prior to a scheduled visit or within 2 days after treatment application. Participating veterinarians and owners were required to collect details on any suspected adverse events throughout the study.

Regular follow-up procedures in weeks 2, 4, 8, and 12 after initial treatment included re-examinations of dogs to document health status and any changes in skin and hair lesions for dogs that originally presented with FAD, followed by parasite counts. In weeks 4 and 8, dogs in the positive control group were retreated after these procedures. Ticks were removed gently with forceps; counted; and categorized as “live or “dead”; ticks were subsequently sent for microscopic identification of the species.

The percentage reduction of ticks in initially infested dogs and percentage reduction of fleas in flea-infested households were calculated for weeks 2, 4, 8, and 12 compared with baseline for both treatments according to the following formula:

$$\text{Reduction}\;\left[\%\right]=\frac{{\overset{¯}{x}}_{\text{pre}-\text{treatment}}-{\overset{¯}{x}}_{\text{post}-\text{treatment}}}{{\overset{¯}{x}}_{\text{pre}-\text{treatment}}}\cdot 100$$

where ${\overset{¯}{x}}_{\text{pre}-\text{treatment}}$ is the geometric mean number of live ticks or fleas at baseline (day 0) and ${\overset{¯}{x}}_{\text{post}-\text{treatment}}$ is the geometric mean number of live ticks or fleas post-treatment (week 2, 4, 8, and 12).

For each visit, non-inferiority of the percentage of parasite-free households was investigated in the Bravecto™ (fluralaner) group compared with the Frontline™ (fipronil) group. The Farrington-Manning test [7] of non-inferiority for the risk difference was used with a level of significance of α=0.025 and a tolerated difference of δ=0.15.

All data for statistical analysis (SAS Institute Inc., Cary, NC, USA, release 9.2) were entered into a computer using the double data entry technique with subsequent comparison of data sets and a plausibility check for missing values, entry errors, and implausible entries.

## Results

The study population included dogs in households in Germany, France, and Spain. Overall, 176 flea-infested households (115 treated with Bravecto™ (fluralaner) and 61 treated with Frontline™ (fipronil)) and 162 tick-infested dogs (108 treated with Bravecto™ (fluralaner) and 54 with Frontline™ (fipronil)) completed the study. Dogs had a mean age of 4.6 years (range 10 weeks to 15 years) and a mean body weight of 19.9 kg (range 2.2 to 59.8 kg); 46% were males (13% of males were neutered) and 54% were females (17% of females were neutered). Breeds represented by more than ten dogs included: Great Anglo-French Hound, English Setter, Spanish Greyhound, Brittany, Beagle, Yorkshire Terrier, Dachshund, Fawn Brittany Basset, Cavalier King Charles Spaniel, and Maltese. Additional population characteristics were recorded (Table 1).

**Table 1:** Demographics of enrolled dog population

	**Bravecto™ (fluralaner)**	**Frontline™ (fipronil)**
	**n = 325**	**n = 154**
Hair length:		
Short	139 (42.8%)	66 (42.9%)
Moderate	137 (42.2%)	64 (41.6%)
Long	49 (15.1%)	24 (15.6%)
Living conditions:		
Inside	84 (25.8%)	44 (28.6%)
Outside	155 (47.7%)	59 (38.3%)
Inside and outside	86 (26.5%)	51 (33.1%)
Number of dogs in the household:		
	n = 144	n = 70
1	76 (52.8%)	32 (45.7%)
2	19 (13.2%)	14 (20.0%)
3	11 (7.6%)	8 (11.4%)
4	11 (7.6%)	8 (11.4%)
5	27 (18.8%)	8 (11.4%)

At baseline (day 0), the mean tick count/dog was 6.5 (range 1–57) and 6.1 (range 1–60) on dogs in the Bravecto™ (fluralaner) and Frontline™ (fipronil) groups, respectively. The most prevalent tick species identified at baseline (day 0) were *Rhipicephalus sanguineus* group ticks (34.8%), followed by *Ixodes hexagonus* (25.4%), *Ixodes ricinus* (25.2%), *Dermacentor reticulatus* (9.6%), *Ixodes* spp. larvae (4.0%), and *Ixodes* spp. nymphs (1.0%). The mean flea count/household was 41.8 (range 0–254 per dog) and 38.1 (range 0–176 per dog) in the Bravecto™ (fluralaner) and Frontline™ (fipronil) groups, respectively (Tables 2 and 3).

**Table 2:** **Household flea infestations before treatment with Bravecto™ (fluralaner) or Frontline™ (fipronil)**

	**Germany**		**France**		**Spain**	
	No. of HHs^*^	**No. of fleas (mean ± std)**	No. of HHs^*^	**No. of fleas (mean ± std)**	No. of HHs^*^	**No. of fleas (mean ± std)**
Bravecto^TM^ (fluralaner)	35	695	52	3056	28	1059
		(19.9 ± 23.0)		(58.8 ± 92.7)		(37.8 ± 38.2)
Frontline^TM^ (fipronil)	17	261	27	1280	17	783
		(15.4 ± 17.2)		(47.4 ± 59.2)		(46.1 ± 44.0)

^*^HHs = households.

**Table 3:** **Dog tick infestations before treatment with Bravecto™ (fluralaner) or Frontline™ (fipronil)**

	**Germany**		**France**		**Spain**	
	**No. of dogs**	**No. of ticks (mean ± std)**	**No. of dogs**	**No. of ticks (mean ± std)**	**No. of dogs**	**No. of ticks (mean ± std)**
Bravecto^TM^ (fluralaner)	26	186	16	164	66	353
		(7.2 ± 8.3)		(10.3 ± 16.7)		(5.3 ± 2.9)
Frontline^TM^ (fipronil)	13	73	11	98	30	160
		(5.6 ± 7.9)		(8.9 ± 17.4)		(5.3 ± 3.1)

Flea-control efficacy in households was higher in Bravecto™ (fluralaner) treated dogs and was 99.2% or higher at all time points (Table 4). Tick-control efficacy on individual dogs was higher in Bravecto™ (fluralaner) treated dogs in weeks 2 and 4. In week 8, Frontline™ (fipronil) efficacy was slightly higher at 100% compared with 99.7% for Bravecto™ (fluralaner). Both treatment groups had a tick efficacy of 100% at week 12 (Table 5).

**Table 4:** Flea control efficacy calculated using household flea counts

	**Bravecto**^**TM **^**(fluralaner)**	**Frontline**^**TM **^**(fipronil)**
Week 2	99.2%	94.1%
Week 4	99.8%	93.0%
Week 8	99.8%	96.0%
Week 12	99.9%	97.3%

**Table 5:** Tick control efficacy calculated using tick counts on individual dogs

	**Bravecto**^**TM **^**(fluralaner)**	**Frontline**^**TM **^**(fipronil)**
Week 2	99.9%	97.6%
Week 4	99.9%	93.8%
Week 8	99.7%	100%
Week 12	100%	100%

The percentage of households free of fleas (top of Table 6, superiority with p < 0.025) was higher after Bravecto™ (fluralaner) treatment compared with Frontline™ (fipronil) treatment at all time points. The percentage of households with Bravecto™ (fluralaner) treated dogs that were free of ticks was higher at all time points compared with households with Frontline™ (fipronil)-treated dogs (bottom of Table 6, non-inferiority with p < 0.0024) except for week 12 when both groups were 100% free of ticks.

**Table 6:** Percentage of households free of fleas or ticks

	**Bravecto™ (fluralaner)**	**Frontline™ (fipronil)**	**p value**	**Lower 97.5% 1-sided C.I.**^**a**^
Fleas				
Week 2	89.57%	62.30%	<0.0001	0.1498
Week 4	94.87%	63.93%	<0.0001	0.1916
Week 8	95.65%	70.49%	<0.0001	0.1416
Week 12	97.39%	81.97%	<0.0001	0.0586
Ticks				
Week 2	97.67%	89.47%	0.0006	-0.0575
Week 4	97.67%	84.21%	0.0001	-0.0175
Week 8	97.67%	94.74%	0.0024	-0.0953
Week 12	100.0%	100.0%	n/a^b^	n/a^b^

^a^Result greater than -0.15 required to declare significant non-inferiority. Result greater than 0 required to declare significant one-sided superiority with α = 0.025.

^b^No statistical test as all households were free of ticks at week 12.

n/a: not applicable.

There were 35 (10.8%) Bravecto™ (fluralaner)-treated dogs and 18 (11.7%) Frontline™ (fipronil)-treated dogs with clinical signs of FAD at inclusion in the study. In the Bravecto™ (fluralaner) group, 85.7% (30 of 35) of these dogs were evaluated at each time point as showing no clinical signs of FAD until the end of the study, while in the Frontline™ (fipronil) group, only 55.6% (10 of 18) had no clinical signs of FAD.

Overall, 8 adverse events reported during the entire study period of 12 weeks were considered to be possibly related to the administered treatment, with 4 reported in each treatment group despite the 1:2 allocation ratio. There were 2 dogs (0.5%) with vomiting/diarrhea and 2 dogs (0.5%) with appetite loss among the 383 dogs in the Bravecto™ (fluralaner)-treated group; all of these dogs recovered from their clinical signs and remained in the study. In the 178 Frontline™ (fipronil)-treated dogs, 3 dogs (1.7%) developed alopecia and crusts in the dorsal lumbo-sacral area and 1 dog (0.6%) developed intense pruritus. All of these dogs remained in the study; 3 recovered and 1 had ongoing clinical signs at the conclusion of the study.

## Discussion

A single orally administered Bravecto™ (fluralaner) treatment at the recommended dose effectively controlled flea and tick infestations in client-owned dogs for 12 weeks under natural infestation challenge. Bravecto™ (fluralaner) is the first orally administered ectoparasiticide to demonstrate this extended period of efficacy against both fleas and ticks on dogs following a single dose. A single Bravecto™ (fluralaner) treatment was significantly non-inferior to 3 sequential Frontline™ (fipronil) treatments for controlling fleas and ticks on dogs, as shown by the lower 97.5% confidence limit > -0.15 at all time points (Table 6). Furthermore, Bravecto™ (fluralaner) treatment was demonstrated to be superior to Frontline™ (fipronil) treatment in the percentage of flea-free households in the study, as shown by the lower 97.5% confidence limit > 0 at all time points (Table 6). Therefore, these efficacy results are consistent with previously reported *in vitro* comparative insect neuronal membrane receptor binding results reported for fluralaner and fipronil [5].

Bravecto™ (fluralaner) posted higher results for flea-control efficacy at every time point (Table 4) and for tick-control efficacy at every time point except for weeks 8 and 12, where the result was close to 100% for both treatments (Table 5). The tick efficacy of Bravecto™ (fluralaner) remained close to 100% over the entire 12 week study period. This is a unique duration of effect for a single orally administered acaricide.

A concern of veterinarians in clinical practice is patient safety for new treatments. Considering the entire study period of 12 weeks, only 4 of the 383 (1.0%) Bravecto™ (fluralaner)-treated dogs in the study had an adverse event and these were exclusively transient gastrointestinal-related events including vomiting and appetite loss. The 178 Frontline™ (fipronil)-treated dogs also had 4 adverse events (2.2%) that were primarily dermal, as might be expected with a topical treatment.

Poor compliance rates with required monthly retreatment protocols for existing ectoparasite therapeutic options are a potential factor in observed reduced ectoparasite treatment efficacy [2]. Bravecto™ (fluralaner) is effective over a 12 week retreatment interval compared to a standard monthly treatment. There is no need to retreat with Bravecto™ (fluralaner), which therefore should offer increased compliance compared with recommended monthly treatments.

Bravecto™ (fluralaner)-treated dogs demonstrated a very strong recovery rate from clinical signs of FAD, with 85.7% of these dogs showing immediate resolution of all clinical signs compared with their skin lesions on entry into the study. Although fleas must feed to be exposed to systemic Bravecto™ (fluralaner) treatment, the degree of flea control efficacy achieved led to the elimination of clinical signs of FAD at a higher rate than observed with topical Frontline™ (fipronil) treatment.

## Conclusions

A single oral dose of Bravecto™ (fluralaner) administered to dogs in a chewable tablet is highly effective for 12 weeks against naturally acquired flea and tick infestations on client-owned dogs under field conditions. A single oral dose of Bravecto™ (fluralaner) is significantly non-inferior (ticks) and superior (fleas) to three doses of topical Frontline™ (fipronil) administered over the same period. Bravecto™ (fluralaner) is safe and well tolerated and the duration of activity offers a more convenient treatment over monthly flea and tick control treatments with a potential compliance advantage.

## Competing interests

All of the authors are employees of MSD Animal Health.

## Authors’ contributions

NR, EZ and RKAR authored the study design and protocol. The study was conducted by NR and EZ completed the statistical calculations. All authors revised and approved the final version.

## References

[B1] Coles TB Dryden MW Insecticide/acaricide resistance in fleas and ticks infesting dogs and cats Parasit Vectors 2014 7 8 10.1186/1756-3305-7-8 24393426 PMC3891977

[B2] Beck S Schein E Baldermann C von Samson-Himmelstjerna G Kohn B Tick infestation and tick prophylaxis in dogs in the area of Berlin/Brandenburg – results of a questionnaire study Berl Munch Tierarztl Wochenschr 2013 126 69 76 23367671

[B3] Ozoe Y Asahi M Ozoe F Nakahira K Mita T The antiparasitic isoxazoline A1443 is a potent blocker of insect ligand-gated chloride channels Biochem Biophys Res Commun 2010 391 744 749 10.1016/j.bbrc.2009.11.131 19944072

[B4] European Commission, Community register of veterinary medicinal products, Product information Bravecto Annex 1 Summary of product characteristics 2014 Bruxelles http://ec.europa.eu/health/documents/community-register/html/v158.htm

[B5] Gassel M Wolf C Noack S Williams H Ilg T The novel isoxazoline ectoparasiticide fluralaner: Selective inhibition of arthropod γ-aminobutyric acid- and L-glutamate-gated chloride channels and insecticidal/acaricidal activity Insect Biochem Mol Biol 2014 45 111 124 24365472 10.1016/j.ibmb.2013.11.009

[B6] Marchiondo AA Holdsworth PA Green P Blagburn BL Jacobs DE World Association for the Advancement of Veterinary Parasitology (WAAVP) guidelines for evaluating the efficacy of parasiticides for the treatment, prevention and control of flea and tick infestation on dogs and cats Vet Parasitol 2007 145 332 344 10.1016/j.vetpar.2006.10.028 17140735

[B7] Farrington CP Manning G Test statistics and sample size formulae for comparative binomial trials with null hypothesis of non-zero risk difference or non-unity relative risk Stat Med 2009 9 1447 1454 2281232 10.1002/sim.4780091208

